# Physicians and AI in healthcare: insights from a mixed-methods study in Poland on adoption and challenges

**DOI:** 10.3389/fdgth.2025.1556921

**Published:** 2025-03-14

**Authors:** Ewelina Kowalewska

**Affiliations:** Department of Management, Kozminski University, Warsaw, Poland

**Keywords:** artificial intelligence (AI), healthcare professionals, physician attitudes, systemic review, Polish healthcare system

## Abstract

**Introduction:**

Understanding healthcare professionals’ attitudes towards artificial intelligence (AI) in medicine is crucial for improving patient care and clinical practice. This study combines a systematic review and a survey targeting Polish physicians to explore these attitudes. While many healthcare professionals express enthusiasm and readiness for AI integration, others remain skeptical due to concerns about reliability, ethical implications, and legal accountability. The systematic review highlighted AI's potential benefits, such as improved diagnostic accuracy and workflow efficiency, alongside challenges like data privacy and the need for validation in atypical scenarios.

**Materials and methods:**

This study combines insights from a systematic review and a targeted survey to assess healthcare professionals’ attitudes toward AI. The survey focused on Polish physicians, a group uniquely positioned to provide insights due to their healthcare system's specific challenges.

**Results:**

The survey revealed optimism among Polish physicians (n86), with 68% ready to adopt AI tools, but underscored the necessity of tailored education and clear implementation guidelines.

**Discussion:**

This study provides valuable insights into the dual narrative of optimism and skepticism surrounding AI in healthcare, emphasizing the importance of addressing barriers to maximize its benefits globally.

## Introduction

In the rapidly evolving landscape of healthcare, the integration of artificial intelligence (AI) stands as a transformative force, promising unparalleled advancements in diagnostics, treatment protocols, and patient care. As the digital age continues to redefine traditional medical practices, the attitudes and perceptions of physicians toward AI technologies emerge as pivotal determinants shaping the future of healthcare delivery.

AI systems offer intelligent solutions to alleviate the burden on clinical staff in healthcare systems facing increasing saturation. This saturation is driven by factors such as an aging population, a rise in chronic diseases, and limited healthcare resources. Unlike simple technological interventions, AI not only manages data but also provides suggestions and recommendations that directly influence clinical decision-making processes (1, 2). These systems hold particular promise in addressing challenges unique to resource-constrained healthcare environments, such as optimizing workflows and improving diagnostic accuracy.

Despite its positive potential, the integration of AI in healthcare elicits mixed attitudes and sentiments among healthcare professionals. This ambivalence reflects the complex interplay of factors shaping physician perspectives on AI, ranging from concerns about its impact on clinical autonomy and reliability to enthusiasm for its potential to enhance diagnostic accuracy and patient outcomes. Concerns about ethical accountability, data privacy, and the role of AI in atypical clinical scenarios further complicate acceptance.

Currently, the adoption of clinical AI remains relatively limited, influenced by factors such as reluctance to change, misperceptions, and knowledge gaps among physicians (3, 4). As key representatives among the primary adopters and operators of AI systems, physicians play crucial roles in integrating AI into clinical practice and management. Thus, physicians’ perspectives must be thoroughly explored and understood. Additionally, AI-driven changes will inevitably impact medical students as the future generation of physicians, necessitating research to develop effective educational and health policies.

While there is a growing body of evidence regarding physician and medical student attitudes toward AI, most research has been conducted in developed, Western countries (5, 6). To address this geographical bias and understand the perspectives of healthcare professionals in different contexts, this study focuses on Polish physicians. The Polish healthcare system is characterized by public funding, significant workload challenges, and rapid digitalization—factors that create a unique environment for AI adoption.

The Polish healthcare system offers a unique perspective compared to other resource-constrained systems globally for several reasons. Firstly, it operates on an insurance-based model where every citizen is entitled to health protection financed through public funds. This model ensures equal access to medical services for all residents, regardless of their financial situation (7). Secondly, Poland is undergoing a process of deinstitutionalization, which means that healthcare services are increasingly available in local environments rather than just in large hospitals. This approach aims to improve the quality of life for the elderly and those with mental health issues (8). Additionally, Poland has strategic development plans for its healthcare system, such as the “Healthy Future” strategy for 2021–2027, with a perspective extending to 2030. This plan aims to create a friendly, modern, and efficient health protection system (9). Furthermore, Poland strives to ensure equal access to high-quality healthcare services for all citizens. This is crucial in the context of limited resources, as it allows for the effective utilization of available means (10).

Comparable systems can be found in countries like Canada, the Netherlands, and the United Kingdom, where an insurance-based model also dominates, ensuring equal access to healthcare. Future research could consider these countries to verify if the conclusions align with the findings in Poland.

To explore these perspectives, a two-stage study was conducted. First, a systematic review identified key trends and knowledge gaps in physician attitudes toward AI globally. Insights from the review informed the design of a targeted questionnaire distributed among physicians in Poland. This approach aimed to gather comprehensive data on their knowledge, perceptions, and readiness to integrate AI into clinical practice. By bridging the gap in existing research, this study contributes valuable insights to the global discourse on AI adoption in healthcare.

## Materials and methods

This study follows a two-stage process, beginning with a systematic review to appraise existing literature on physicians’ preferences regarding artificial intelligence (AI). Insights from the review were subsequently used to design a survey targeting Polish physicians to capture their specific perspectives.

**Figure 1:**
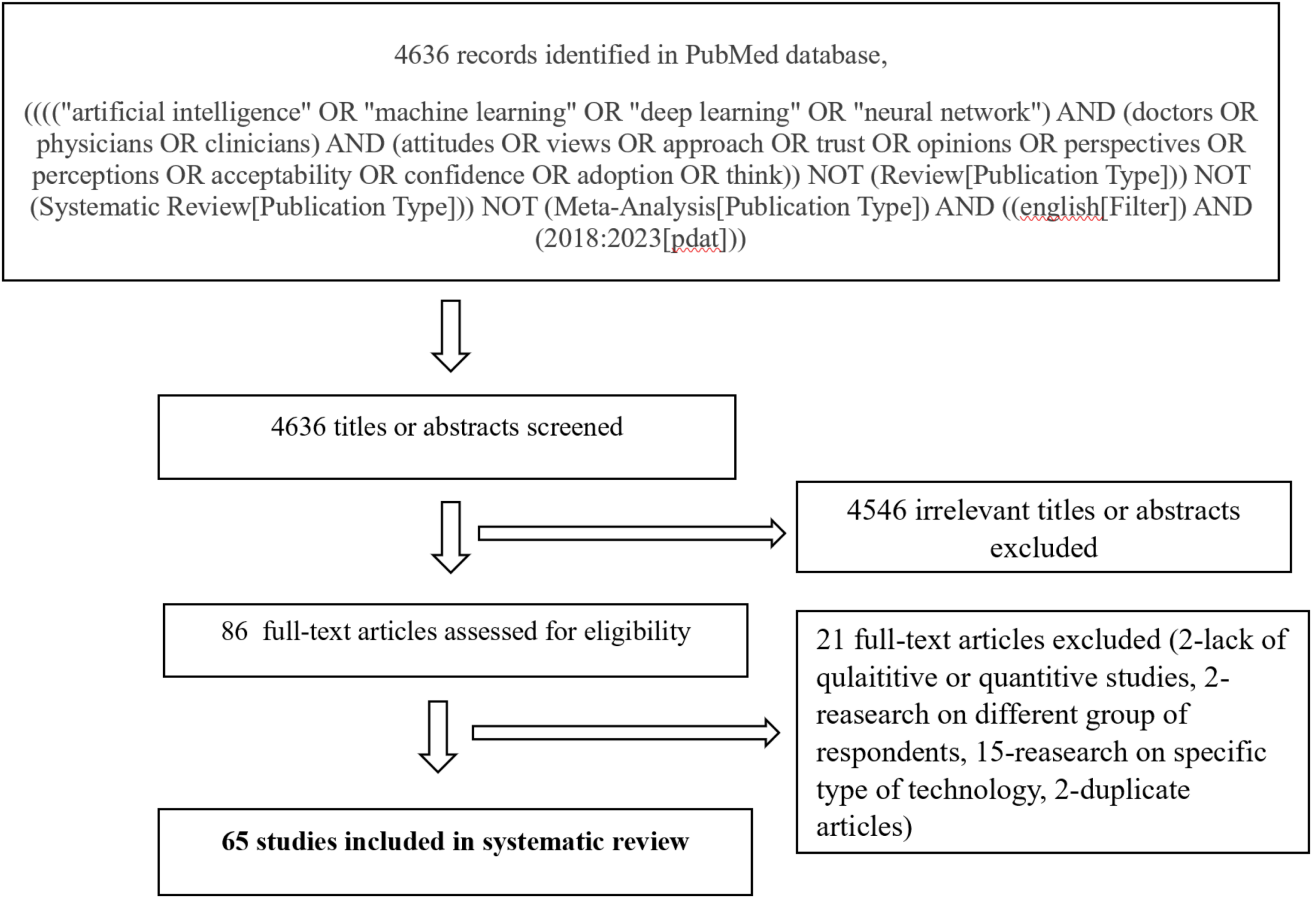
PRISMA flow chart.

The initial search string for this systematic review included studies appraising physicians’ preferences regarding AI. Articles were limited to those published in English between January 1, 2018, and June 12, 2023. The search, conducted between June and July 2023, identified 4,636 records in the PubMed database. Figure 1 illustrates the PRISMA flowchart, detailing the various stages of the systematic review process.

In this review, AI was specifically defined as technology designed to automate intelligent actions within clinical environments, thereby supporting clinical decision support systems and enhancing physician-mediated care. Consumer-oriented products like wearable devices were excluded from this definition to maintain a focus on technologies directly involved in clinical practice.

## Methodology

The systematic review was conducted by the author in accordance with the PRISMA (Preferred Reporting Items for Systematic Reviews and Meta-Analyses) guidelines to ensure a transparent and reproducible review process. The review followed a rigorous selection process based on predefined inclusion and exclusion criteria. The primary aim was to identify empirical studies that focused on physicians’ preferences toward the adoption of artificial intelligence (AI) in healthcare settings, rather than clinical trials, technical development, or expert opinions on specific AI tools. This allowed the study to maintain focus on how physicians perceive, accept, and interact with AI technologies within clinical practice.

### Selection criteria

Inclusion criteria were applied to studies that:

• Focused on physicians’ attitudes, perceptions, or adoption of AI technologies in healthcare, with an emphasis on attitudes, opinions, confidence, trust, or adoption related to AI systems and their integration into clinical workflows.
• Included empirical data gathered through qualitative or quantitative research methods, such as surveys, interviews, or focus groups.
• Were published between 2018 and 2023, and in English, to ensure the inclusion of up-to-date studies with high relevance to the current state of AI in healthcare.
• Targeted healthcare professionals, particularly doctors, physicians, and clinicians, in the context of their engagement with AI technologies, either in diagnostics, treatment planning, or administrative tasks.

Exclusion criteria were applied to studies that:

• Were review articles, systematic reviews, or meta-analyses, as these do not provide new, original empirical data.
• Focused on clinical trials or the technical development of AI tools without addressing physician perspectives or preferences.
• Were theoretical or opinion-based articles, such as editorials or expert opinions, which did not involve empirical data related to physician attitudes.
• Only involved medical students or non-clinical populations, as the focus of this review was on active healthcare practitioners.
• Did not include physician perspectives but instead discussed the potential applications of AI from a technological or clinical perspective.

## Study selection process

The initial database search yielded 4,636 articles. These articles were systematically screened for relevance, based on the criteria above. Following the initial screening, a significant portion of studies was excluded due to irrelevance, primarily because they described clinical trials, technical development of AI tools, or expert opinions rather than focusing on physicians’ preferences toward AI. The remaining studies were subjected to full-text review, and final selection was based on whether the study met all of the inclusion criteria and adhered to the scope of this review.

Supplementary materials accompanying this manuscript include the PRISMA Checklist to document the systematic review process, a comprehensive list of all studies reviewed (indicating which were accepted or rejected, with reasons for exclusion), and the English version of the survey questionnaire used to gather physician perspectives in the subsequent primary research phase. These materials are provided to ensure full transparency and allow for the reproducibility of the study's methodology.

## Findings from the literature review

The review identified a variety of benefits associated with AI integration in healthcare. Studies emphasized that AI will enhance rather than replace the roles of physicians, as noted by van der Zander et al. (11) and AlZaabi et al. (12). Improved quality of medical services was highlighted as a key benefit by van der Zander et al. (11), while significant reductions in diagnostic errors were reported by Coppola et al. (13) and Khafaji et al. (14). Additionally, AI was shown to optimize workflow by streamlining processes and reducing repetitive tasks, as demonstrated by Coppola et al. (13) and Horsfall et al. (15). The potential for AI to create new roles within healthcare was noted by AlZaabi et al. (12), and its ability to analyze large datasets was emphasized by Oh et al. (16). Furthermore, von Wedel et al. (17) underscored AI's efficiency in reducing diagnostic time, highlighting its potential time-saving benefits.

However, several barriers to AI implementation were also identified. Oh et al. (16) highlighted the lack of diagnostic support in atypical cases, while studies by Buck et al. (18) and Chen et al. (19) suggested that AI might alter traditional physician roles and potentially disrupt patient-physician relationships. Buck et al. (18) also noted instances where AI implementation led to higher diagnostic errors. Concerns about the inappropriate use of patient data were raised by Buck et al. (18), and the need for clear legal frameworks to assign responsibility for AI-driven decisions was emphasized by AlZaabi et al. (12) and Horsfall et al. (15).

For effective AI integration, several prerequisites were outlined. Jungmann et al. (20) emphasized the importance of regular validation to ensure algorithm reliability, while Pangti et al. (21) and Pecqueux et al. (22) advocated for mandatory specialized training programs for physicians. Strohm et al. (23) underscored the necessity of demonstrating AI's clinical value before its implementation.

The general insights from the literature further highlight the diverse perspectives on AI adoption. While some studies indicated that AI might alter physician roles or potentially replace certain functions (Quirine et al.; Buck et al.; Mawya et al.; Chen et al.), other research, such as that by Coppola et al. and Shelmerdine et al., consistently affirmed that AI would not replace physicians but rather support them. Commonly cited benefits included improved service quality, reduced diagnostic errors, and significant time savings for physicians (Quirine et al., Coppola et al., Horsfall et al.). Strohm et al. (23) emphasized that successful AI integration requires structured approaches, including collaboration between radiologists and clinicians. Diprose et al. noted that trust in AI solutions is heavily dependent on physician involvement in their development and validation. Education and training on AI technologies were highlighted as critical by Cobianchi et al., Pangti et al., and SalAlzaabi et al., with most respondents expressing willingness to adopt AI if it was supported by international guidelines or included in formal training programs (12).

The literature review was conducted primarily to gather data for the survey questionnaire and focused on articles published between 2018 and 2023. The literature review will be continued to address gaps in future publications. However, for the purpose of creating the survey questionnaire, the information obtained from the articles was sufficient.

## Updated perspectives on AI in healthcare

Additionally, to further enhance the literature review for this manuscript, the authors incorporated more recent articles published in 2024 and 2025. This approach ensured the inclusion of the latest findings to provide a more comprehensive foundation for the study.

One of the key insights from these recent studies is the evolving role of medical specialists within an AI-augmented healthcare system. For instance, Sedaghat (24) explores how AI-based imaging technologies, including ChatGPT-driven applications, are transforming radiology. The study suggests that AI could automate routine tasks, such as the preliminary analysis of x-rays and MRIs, leading to faster diagnostic processes and reduced workload for radiologists. However, while automation is expected to streamline standard procedures and optimize diagnostic workflows, complex imaging cases will still require expert human oversight. Rather than replacing radiologists, AI is poised to enhance their efficiency, allowing them to focus on high-complexity diagnostics and patient-centered decision-making.

Another study published in 2025 (25) examines physicians’ attitudes toward AI integration, particularly in AI-assisted triage for MRI brain scans. A survey of 133 clinicians in the UK found that 71% preferred AI-assisted triage over traditional chronological approaches. Moreover, the use of visual explanations, such as heatmaps, significantly increased clinician confidence, with 60% of participants reporting higher trust in AI-generated diagnostic suggestions. These findings reinforce the growing acceptance of AI in radiology, demonstrating its potential to enhance workflow efficiency and diagnostic accuracy.

Beyond radiology, AI's integration into primary care has also been explored in recent research. Allen et al. (26) conducted a mixed-methods study assessing primary care physicians’ (PCPs) attitudes toward AI-driven decision support tools. While PCPs acknowledged AI's role in improving efficiency—particularly in disease screening, chronic disease management, and administrative support—they also expressed concerns about equity in healthcare access and the potential impact on the doctor-patient relationship. The study highlights the importance of addressing challenges such as algorithmic bias, workflow integration, and regulatory barriers to ensure that AI complements, rather than disrupts, primary care practices.

These updated perspectives provide valuable insights into the evolving landscape of AI in medicine, highlighting the need for a balanced approach—one that harnesses AI's capabilities while preserving the essential role of human expertise in patient care. These findings emphasize the importance of overcoming existing challenges and establishing robust frameworks to ensure the effective and ethical integration of AI in healthcare.

## Questionnaire survey

A web-based questionnaire was developed based on the findings from the systematic review to capture the perspectives of Polish physicians on AI implementation in healthcare.

Polish physicians operate under significant workload and resource constraints, creating both challenges and opportunities for adopting AI solutions. These specific conditions made Polish physicians an ideal study group to analyze the readiness and perceptions of AI adoption in clinical practice.

The draft questionnaire underwent a pilot phase among a group of physicians, allowing for adjustments based on the feedback collected. The final version of the survey consisted of 14 questions: 7 demographic questions (age, gender, professional experience, medical specialty, workplace, etc.) and 6 questions related to AI preferences. These included items on attitudes towards using AI, perceived benefits and challenges of AI in medical practice, and whether AI could replace physicians. Additionally, a Likert scale section assessed statements on AI, such as the importance of regular validation and reliability checks, the legal accountability of AI recommendations, and the need for specialized training before AI implementation.

The choice of demographic questions was carefully considered to ensure a comprehensive understanding of the respondents’ backgrounds and their potential influence on attitudes towards AI.

### Age

Age was included to identify potential differences in attitudes towards AI among various age groups. Younger physicians may be more open to new technologies, whereas older physicians might exhibit more skepticism.

### Professional experience

Years of professional experience were assessed to explore how experience level might affect readiness to adopt AI. More experienced physicians might have established workflows that are less flexible to change.

### Workplace location (urban/rural)

The location of the workplace was included to account for differences in resource availability and technology access between urban and rural settings. Physicians in urban areas might have better access to advanced technologies compared to their rural counterparts.

### Gender

Gender was considered to examine if there are any gender-based differences in the perception and acceptance of AI in medical practice.

These demographic groups were compared using the chi-square test of independence. The analysis did not reveal any statistically significant differences between the demographic groups in terms of AI usage in daily practice.

The rationale for using a Likert scale was based on several key factors:

### Measurement of attitudes and beliefs

Likert scales allow for a nuanced measurement of respondents’ attitudes and beliefs, capturing varying degrees of agreement or disagreement with specific statements.

### Complexity of the topic

Given the multifaceted nature of AI in medicine, Likert scales are well-suited to assess subtle differences in opinions and perceptions that might not be captured through simple yes/no questions.

### Ease of data analysis

Data from Likert scales are conducive to statistical analysis, facilitating the comparison of responses across different demographic groups and identifying significant patterns.

### Prevalence in quantitative research

Likert scales are widely used in quantitative research, making the results more comparable with existing studies and easier to contextualize within the broader scientific literature.

The final version of the survey was distributed through multiple channels. On August 8, 2023, a link was included in a newsletter sent to all physicians registered in the Supreme Medical database. The “Supreme Medical Database” refers to the Central Register of Physicians (Centralny Rejestr Lekarzy), managed by the Supreme Medical Chamber (Naczelna Izba Lekarska) in Poland. This database provides essential information about physicians and dentists, including their professional qualifications and practice rights. It is accessible online at https://rejestr.nil.org.pl/?active_id = F44.0&utm_source = chatgpt.com. Subsequently, on August 28, 2023, the link was posted on two prominent Polish healthcare and medicine-focused websites, pulsmedycyny.pl and konsylium24.pl. By November 23, 2023, 86 responses had been received. Data analysis was performed based on these responses.

## Ethical considerations

In accordance with Polish law, survey-based studies that do not involve patient data or interventions are exempt from the requirement of bioethics committee approval. Therefore, this survey did not require formal ethical approval.

## Rationale for the survey

This study aimed to investigate the unique factors influencing Polish physicians’ perceptions and readiness for AI integration. The selection of Polish physicians was motivated by the distinct characteristics of the Polish healthcare system, including its relatively lower healthcare expenditure as a percentage of GDP compared to other EU countries, significant workload challenges, and the rapid adoption of digital healthcare tools such as electronic medical records, e-prescriptions, and e-referrals. These factors provided a rich context for understanding how local conditions shape attitudes towards AI in healthcare, contributing valuable insights to the global discourse on AI adoption in clinical practice.

One limitation of this study is the potential for response bias, as the survey relies on self-reported data, which may lead participants to provide socially desirable answers rather than their genuine opinions about AI. To mitigate this limitation in future research, strategies such as anonymous surveys and complementary qualitative methods, including interviews, could be employed to gain more nuanced and authentic insights.

## Results

### Knowledge, understanding, and attitudes towards AI

The systematic review provided a comprehensive overview of the current state of research on physicians’ preferences for AI in healthcare. Several studies emphasized the potential benefits of AI integration, including enhanced diagnostic accuracy, improved quality of medical services, and streamlined workflows. For example, van der Zander et al. (11) and Coppola et al. (13) highlighted that AI could reduce diagnostic errors and optimize repetitive tasks, enabling physicians to dedicate more time to patient care. Furthermore, AlZaabi et al. (12) and Oh et al. (16) demonstrated AI's capacity to analyze large datasets and create new opportunities within the healthcare sector. Despite these advantages, barriers to adoption were also noted. Concerns included AI's inability to handle atypical clinical cases, potential disruptions to patient-physician relationships, and the absence of clear legal frameworks for accountability in AI-driven decisions (15, 18).

Additionally, the systematic review underscored the importance of structured implementation strategies. These include rigorous validation of AI algorithms, mandatory training programs for healthcare professionals, and evidence demonstrating AI's clinical value prior to widespread adoption. These insights served as a foundation for developing the questionnaire used to explore Polish physicians’ perspectives.

The survey, conducted among 86 Polish physicians, provided detailed insights into their attitudes and readiness for AI adoption in clinical practice. A majority of respondents (68%) expressed optimism about integrating AI into their workflows, with 20% already utilizing AI-based tools. Nonetheless, 9% reported disinterest, while 2% remained undecided. The analysis revealed no significant differences in attitudes toward the use of AI with respect to respondents’ age, gender, or workplace setting. These findings suggest that perceptions of AI integration in healthcare are consistent across demographic and professional groups, indicating a broadly shared perspective on the potential benefits and challenges of AI adoption. Respondents identified key benefits, including improved diagnostic accuracy, significant time savings, and enhanced capability to manage large datasets. These findings align with global trends observed in the systematic review, reflecting a general awareness of AI's advantages.

However, the survey also revealed substantial concerns among respondents. A significant number highlighted issues related to the reliability of AI tools, particularly in managing atypical or complex clinical scenarios. Legal accountability and ethical implications of AI usage were also frequently cited as barriers. Respondents emphasized the necessity of regular validation and monitoring of AI systems, as well as specialized training programs to prepare physicians for AI adoption. The importance of incorporating AI-related guidelines into formal medical education and adhering to international standards was also highlighted as critical for fostering trust and effective usage.

### Perceptions of AI implementation in healthcare

The systematic review and survey both revealed diverse perceptions of AI's role in healthcare. While van der Zander et al. (11) and Coppola et al. (13) emphasized the potential of AI to enhance diagnostic accuracy and reduce errors, other studies highlighted apprehensions. Buck et al. (18) noted concerns regarding ethical and legal accountability, reflecting the broader spectrum of expectations surrounding AI adoption. These findings were mirrored in the survey of Polish physicians, where optimism about AI's transformative potential coexisted with skepticism regarding its reliability and ethical implications.

Survey respondents particularly valued AI's potential to streamline workflows, reduce repetitive tasks, and assist in analyzing large datasets. However, concerns about the technology's limitations in handling atypical cases and the potential for over-reliance were evident. The emphasis on robust validation and education highlights a cautious but hopeful approach to AI integration.

## Synthesis of findings

Overall, the combined findings from the systematic review and the survey highlight a dual narrative of optimism and skepticism towards AI in healthcare. While AI is recognized for its transformative potential—enhancing efficiency, accuracy, and data handling capabilities—significant apprehensions remain regarding its reliability, ethical considerations, and legal accountability. These insights underscore the need for targeted interventions, such as educational initiatives and policy frameworks, to address barriers and ensure the effective integration of AI into clinical practice. To this end, specific strategies can be proposed to enhance the practical value of the findings. For instance, implementing AI-focused educational programs in medical schools and continuous professional development courses can bridge knowledge gaps and build AI literacy among healthcare professionals. Additionally, developing comprehensive policy frameworks that address ethical, legal, and accountability concerns related to AI can foster a trustworthy environment for AI adoption. These frameworks should include clear guidelines for AI validation and usage, as well as mechanisms for regular oversight and updates. Furthermore, establishing collaborative platforms where healthcare providers, AI developers, and policymakers can share insights and experiences will be crucial in aligning AI applications with clinical needs and ensuring their effectiveness and safety. By adopting these strategies, the healthcare community can effectively navigate the challenges posed by AI integration and harness its full potential for improving patient care and clinical workflows.

## Discussion: understanding healthcare professionals’ attitudes towards AI

The insights gleaned from this study provide a nuanced understanding of the complex and evolving attitudes of healthcare professionals towards artificial intelligence (AI) in medicine. These findings highlight the critical need to address both the opportunities and challenges associated with AI integration to effectively harness its potential for improving patient care and clinical workflows.

The systematic review revealed varying levels of engagement, familiarity, and readiness among healthcare professionals regarding AI integration. Studies such as those by Khafaji et al. (14) and Pecqueux et al. (22) demonstrated a significant willingness among respondents to learn and train on AI technologies, indicating a readiness for integration. However, knowledge gaps remain evident, particularly in specialties like surgery, as highlighted by De Simone et al. (27). This underscores the necessity for targeted education and training initiatives to bridge these gaps and enhance AI literacy across medical domains.

Perceptions of AI implementation also exhibit a spectrum of beliefs and expectations. While van der Zander et al. (11) and Coppola et al. (13) emphasized the potential of AI to enhance diagnostic accuracy and reduce errors, other studies, such as those by Buck et al. (18), raised concerns about ethical and legal accountability. This duality is echoed in the survey findings, where Polish physicians expressed optimism about AI's transformative potential but also highlighted apprehensions regarding its reliability and the need for clear legal frameworks.

The unique context of the Polish healthcare system provides additional insights into the factors influencing AI adoption. Operating within a resource-constrained environment characterized by significant workload challenges and rapid digitalization, Polish physicians are well-positioned to benefit from AI technologies. However, their concerns about implementation underscore the importance of tailored strategies that address local conditions. For instance, incorporating AI training into medical education and establishing robust guidelines for AI validation and usage were frequently cited as critical steps by survey respondents. These observations in Poland can also provide valuable insights for other countries with similar healthcare challenges. For example, countries such as Canada, the Netherlands, and the United Kingdom, which also operate on insurance-based healthcare models, could benefit from similar strategies tailored to their unique contexts.

Additionally, the survey's limited scope, focusing exclusively on Polish physicians, reflects the need for further research involving larger and more diverse samples. Expanding the study to include physicians from different specialties and nationalities would allow for a broader understanding of global trends in AI adoption and help identify universal and context-specific factors influencing its implementation. For instance, future research could explore whether healthcare professionals in resource-constrained environments elsewhere share similar attitudes towards AI, potentially leading to global strategies for AI integration in healthcare.

Recent studies from 2024 to 2025 shed light on the evolving role of AI in healthcare, providing evidence that either validates or challenges earlier predictions. While initial enthusiasm centered on AI's capacity to reduce clinician workload and enhance diagnostic accuracy, these newer findings highlight concrete advancements, particularly in AI-assisted triage and automated imaging analysis. Concerns about job displacement, once prominent, have largely been alleviated, with AI increasingly viewed as a supportive tool that augments clinical expertise rather than replaces it. These insights highlight the value of re-evaluating earlier forecasts with up-to-date evidence, ensuring discussions remain relevant to the current landscape of AI adoption in medicine.

The findings of this study underscore the need for future research, emphasizing the importance of considering both local and global healthcare contexts.

A collaborative approach is essential for the successful integration of AI, requiring input from policymakers, educators, and technology developers to create reliable and ethically sound tools that align with the needs of healthcare professionals. By building trust and addressing potential challenges, the medical community can unlock the full potential of AI while minimizing risks. This research contributes to the broader conversation on AI in healthcare, offering a framework for understanding and addressing the diverse perspectives of medical professionals worldwide.

## Data Availability

The raw data supporting the conclusions of this article will be made available in a limited capacity and only after the defense of the doctoral dissertation in which the same data will be used. Further inquiries can be directed to the corresponding author.
