# Supplementary material for: Physicians and AI in healthcare: insights from a mixed-methods study in Poland on adoption and challenges
Source: Front Digit Health. 2025 Mar 14;7:1556921. doi: 10.3389/fdgth.2025.1556921 (PMC11949901; doi:10.3389/fdgth.2025.1556921)
Supplement: Supplementary file 1 [file Table1.docx]

# PRISMA Checklist

The following PRISMA Checklist provides a detailed account of the systematic review conducted in the study 'Physicians and AI in Healthcare: A Mixed-Methods Study Exploring Opportunities and Challenges'.

| Section and Topic | Item | Reported on Page Number |
| --- | --- | --- |
| Title | Title identifies the report as a systematic review | Title |
| Abstract | Structured summary of objectives, methods, results, and conclusions | Abstract |
| Introduction | Rationale explains the context and need for the review | Introduction |
| Introduction | Objectives clearly state the questions being addressed | Introduction |
| Methods | Eligibility criteria for included studies | Materials and Methods |
| Methods | Information sources and search strategy detailed | Materials and Methods |
| Methods | Data extraction and synthesis methods described | Materials and Methods |
| Methods | Risk of bias assessment methods mentioned | Materials and Methods |
| Results | Number of studies included, with reasons for exclusion | Results, PRISMA Flowchart |
| Results | Characteristics of included studies provided | Results |
| Results | Summary of findings (quantitative or qualitative) | Results |
| Discussion | Summary of evidence and implications | Discussion |
| Discussion | Limitations of the review noted | Discussion |
| Discussion | Future research directions suggested | Discussion |
| Funding | Funding sources and potential conflicts of interest disclosed | Acknowledgements |
