# Supplementary material for: Physicians and AI in healthcare: insights from a mixed-methods study in Poland on adoption and challenges
Source: Front Digit Health. 2025 Mar 14;7:1556921. doi: 10.3389/fdgth.2025.1556921 (PMC11949901; doi:10.3389/fdgth.2025.1556921)
Supplement: Supplementary file 2 [file Datasheet1.pdf]

Systemic review

(((("artificial intelligence" OR "machine learning" OR "deep learning" OR "neural network") AND (doctors OR physicians OR clinicians) AND (attitudes OR views OR approach OR trust OR opinions OR perspectives OR perceptions OR acceptability OR confidence OR adoption OR think)) NOT (Review[Publication Type])) NOT (Systematic Review[Publication Type])) NOT (Meta-Analysis[Publication Type]) AND ((english[Filter]) AND (2018:2023[pdat]))

4,636 results

65 studies included in systemic review

1. Surgeons' perspectives on artificial intelligence to support clinical decision-making in trauma and emergency contexts: results from an international survey. DOI: 10.1186/s13017-022-00467-3
2. Artificial intelligence in (gastrointestinal) healthcare: patients' and physicians' perspectives. DOI: 10.1038/s41598-022-20958-2
3. Attitudes Toward Artificial Intelligence Among Radiologists, IT Specialists, and Industry. DOI: 10.1016/j.acra.2020.04.011
4. General Practitioners' Attitudes Toward Artificial Intelligence-Enabled Systems: Interview Study. DOI: 10.2196/28916
5. Artificial intelligence: radiologists' expectations and opinions gleaned from a nationwide online survey. DOI: 10.1007/s11547-020-01205-y
6. Artificial intelligence in paediatric radiology: international survey of health care rofessionals' opinions. DOI: 10.1007/s00247-021-05195-5
7. Acceptability of artificial intelligence among Indian dermatologists. DOI: 10.25259/IJDVL_210_2021
8. Are physicians and medical students ready for artificial intelligence applications in healthcare? DOI: 10.1177/20552076231152167
9. Physician Confidence in Artificial Intelligence: An Online Mobile Survey. DOI: 10.2196/12422
10. Physician understanding, explainability, and trust in a hypothetical machine learning risk calculator. DOI: 10.1093/jamia/ocz229
11. Artificial intelligence in radiology: Are Saudi residents ready, prepared, and knowledgeable? DOI: 10.15537/smj.2022.43.1.20210337
12. The use and future perspective of Artificial Intelligence-A survey among German surgeons. DOI: 10.3389/fpubh.2022.982335
13. Acceptance of clinical artificial intelligence among physicians and medical students: A systematic review with cross-sectional survey. DOI: 10.3389/fmed.2022.990604
14. Artificial intelligence and the future of psychiatry: Insights from a global physician survey. DOI: 10.1016/j.artmed.2019.101753
15. Implementation of artificial intelligence (AI) applications in radiology: hindering and facilitating factors.DOI: 10.1007/s00330-020-06946-y
16. Attitudes of the Surgical Team Toward Artificial Intelligence in Neurosurgery: International 2-Stage Cross-Sectional Survey. DOI: 10.1016/j.wneu.2020.10.171

17. Physicians' preferences and willingness to pay for artificial intelligence-based assistance tools: a discrete choice experiment among german radiologists. DOI: 10.1186/s12913-022-07769-x
18. Machine learning in neurosurgery: a global survey. DOI: 10.1007/s00701-020-04532-1
19. Physical Therapists' Knowledge and Attitudes Regarding Artificial Intelligence Applications in Health Care and Rehabilitation: Cross-sectional Study. DOI: 10.2196/39565
20. Artificial Intelligence and the Future of Primary Care: Exploratory Qualitative Study of UK General Practitioners' Views. DOI: 10.2196/12802
21. Clinician perspectives on machine learning prognostic algorithms in the routine care of patients with cancer: a qualitative study. DOI: 10.1007/s00520-021-06774-w
22. Radiation Oncologists' Perceptions of Adopting an Artificial Intelligence-Assisted Contouring Technology: Model Development and Questionnaire Study. DOI: 10.2196/27122
23. Patients' and Clinicians' Perceived Trust in Internet-of-Things Systems to Support Asthma Self-management: Qualitative Interview Study. DOI: 10.2196/24127
24. Attitudes of medical workers in China toward artificial intelligence in ophthalmology: a comparative survey. DOI: 10.1186/s12913-021-07044-5
25. Knowledge, attitude, and practice of artificial intelligence in emergency and trauma surgery, the ARIES project: an international web-based survey. DOI: 10.1186/s13017-022-00413-3
26. Intensive Care Unit Physicians' Perspectives on Artificial Intelligence-Based Clinical Decision Support Tools: Preimplementation Survey Study. DOI: 10.2196/39114
27. Future Medical Artificial Intelligence Application Requirements and Expectations of Physicians in German University Hospitals: Web-Based Survey. DOI: 10.2196/26646
28. Does AI explainability affect physicians' intention to use AI? DOI: 10.1016/j.ijmedinf.2022.104884
29. Evaluation of pediatric ophthalmologists' perspectives of artificial intelligence in ophthalmology. DOI: 10.1016/j.jaapos.2021.01.011
30. Clinician and computer: a study on doctors' perceptions of artificial intelligence in skeletal radiography. DOI: 10.1186/s12909-022-03976-6
31. Radiologist Preferences for Artificial Intelligence-Based Decision Support During Screening Mammography Interpretation. DOI: 10.1016/j.jacr.2022.06.019
32. UK reporting radiographers' perceptions of AI in radiographic image interpretation - Current perspectives and future developments. DOI: 10.1016/j.radi.2022.06.006
33. Dermatologists' Perspectives on Artificial Intelligence and Augmented Intelligence - A Cross-sectional Survey. DOI: 10.1001/jamadermatol.2021.1685
34. Artificial intelligence in radiology: trainees want more. DOI: 10.1016/j.crad.2022.12.017
35. Assessing Barriers to Implementation of Machine Learning and Artificial Intelligence-Based Tools in Critical Care: Web-Based Survey Study. DOI: 10.2196/41056
36. "Many roads lead to Rome and the Artificial Intelligence only shows me one road": an interview study on physician attitudes regarding the implementation of computerised clinical decision support systems. DOI: 10.1186/s12910-022-00787-8

37. A survey on the future of radiology among radiologists, medical students and surgeons: students and surgeons tend to be more skeptical about artificial intelligence and radiologists may fear that other disciplines take over. DOI: 10.1016/j.ejrad.2019.108742
38. A Nationwide Web-Based Survey of Neuroradiologists' Perceptions of Artificial Intelligence Software for Neuro-Applications in Korea. DOI: 10.3348/kjr.2022.0905
39. Perceptions of Canadian radiation oncologists, radiation physicists, radiation therapists and radiation trainees about the impact of artificial intelligence in radiation oncology - national survey. DOI: 10.1016/j.jmir.2020.11.013
40. Towards successful implementation of artificial intelligence in skin cancer care: a qualitative study exploring the views of dermatologists and general practitioners. DOI: 10.1007/s00403-022-02492-3
41. Professionals' responses to the introduction of AI innovations in radiology and their implications for future adoption: a qualitative study. DOI: 10.1186/s12913-021-06861-y Hospital-wide survey of clinical experience with artificial intelligence applied to daily chest radiographs. DOI: 10.1371/journal.pone.0282123
42. Artificial intelligence in orthopedics: A qualitative exploration of the surgeon perspective. DOI: 10.1097/MD.0000000000034071
43. An international survey on AI in radiology in 1,041 radiologists and radiology residents part 1: fear of replacement, knowledge, and attitude. DOI: 10.1007/s00330-021-07781-5
44. Knowledge, attitude, and practice of artificial intelligence among doctors and medical students in Syria: A cross-sectional online survey. DOI: 10.3389/frai.2022.1011524
45. A Survey on the Use of Artificial Intelligence by Clinicians in Dentistry and Oral and Maxillofacial Surgery. DOI: 10.3390/medicina58081059
46. Artificial intelligence and the future of psychiatry: Qualitative findings from a global physician survey. DOI: 10.1177/2055207620968355
47. Knowledge, attitudes, and practices towards artificial intelligence among young pediatricians: A nationwide survey in France. DOI: 10.3389/fped.2022.1065957
48. How Clinicians Perceive Artificial Intelligence-Assisted Technologies in Diagnostic Decision Making: Mixed Methods Approach. DOI: 10.2196/33540
49. Artificial Intelligence for Radiotherapy Auto-Contouring: Current Use, Perceptions of and Barriers to Implementation. DOI: 10.1016/j.clon.2023.01.014
50. Physicians' attitudes and knowledge toward artificial intelligence in medicine: Benefits and drawbacks. DOI: 10.1016/j.heliyon.2023.e14744
51. Perceptions of Artificial Intelligence Among Healthcare Staff: A Qualitative Survey Study. DOI: 10.3389/frai.2020.578983
52. Exploring Physician Perspectives on Using Real-world Care Data for the Development of Artificial Intelligence-Based Technologies in Health Care: Qualitative Study. DOI: 10.2196/35367
53. Differences in Knowledge and Perspectives on the Usage of Artificial Intelligence Among Doctors and Medical Students of a Developing Country: A Cross-Sectional Study. DOI: 10.7759/cureus.21434
54. Perceptions of Canadian vascular surgeons toward artificial intelligence and machine learning. DOI: 10.1016/j.jvscit.2022.06.018
55. "Nothing works without the doctor:" Physicians' perception of clinical decision-making and artificial intelligence. DOI: 10.3389/fmed.2022.1016366

56. A survey of clinicians on the use of artificial intelligence in ophthalmology, dermatology, radiology and radiation oncology. DOI: 10.1038/s41598-021-84698-5
57. Attitudes of optometrists towards artificial intelligence for the diagnosis of retinal disease: A cross-sectional mail-out survey. DOI: 10.1111/opo.13034
58. Attitudes Towards Artificial Intelligence Among Dermatologists Working in Saudi Arabia. DOI: 10.5826/dpc.1301a35
59. Exploring the experiences and views of doctors working with Artificial Intelligence in English healthcare; a qualitative study. doi: 10.1371/journal.pone.0282415. eCollection 2023
60. Attitudes of Anesthesiologists toward Artificial Intelligence in Anesthesia: A Multicenter, Mixed Qualitative-Quantitative Study. DOI: 10.3390/jcm12062096
61. Acceptance of the Use of Artificial Intelligence in Medicine Among Japan's Doctors and the Public: A Questionnaire Survey. DOI: 10.2196/24680
62. Health Care Employees' Perceptions of the Use of Artificial Intelligence Applications: Survey Study. DOI: 10.2196/17620
63. Web-based study on Chinese dermatologists' attitudes towards artificial Intelligence DOI: 10.21037/atm.2019.12.102
64. Physician perspectives on integration of artificial intelligence into diagnostic pathology. DOI: 10.1038/s41746-019-0106-0
65. Physician sentiment toward artificial intelligence (AI) in colonoscopic practice: a survey of US gastroenterologists. DOI: 10.1055/a-1223-1926

21 full-text aricles exluded

1. Will AI Replace Ophthalmologists? DOI: 10.1167/tvst.9.2.2 – lack of qualititive or quantitive studies
2. Trust in artificial intelligence for medical diagnoses. DOI: 10.1016/bs.pbr.2020.06.006 – researach on different group of responders
3. The Impact of Cultural Dimensions of Clinicians on the Adoption of Artificial Intelligence in Healthcare. PMID: 35062809 [Indexed for MEDLINE] - lack of qualititive or quantitive studies
4. Resident Physicians' Perceptions of Diagnostic Radiology and the Declining Interest in the Specialty. DOI: 10.1016/j.acra.2020.01.016 – reasearch on specific type of technology
5. A Physician-in-the-Loop Approach by Means of Machine Learning for the Diagnosis of Lymphocytosis in the Clinical Laboratory. DOI: 10.5858/arpa.2021-0044-OA – reasearch on specific type of technology
6. Artificial Intelligence and Its Effect on Dermatologists' Accuracy in Dermoscopic Melanoma Image Classification: Web-Based Survey Study. DOI: 10.2196/18091 – reasearch on specific type of technology

7. The views of physicians and nurses on the potentials of an electronic assessment system for recognizing the needs of patients in palliative care. DOI: 10.1186/s12904-020-00554-9 - reasearch on specific type of technology
8. An exploration of expectations and perceptions of practicing physicians on the implementation of computerized clinical decision support systems using a Qsort approach. DOI: 10.1186/s12911-022-01933-3 – reasearch on specific type of technology
9. A survey of current practices, attitudes and demands of anaesthesiologists regarding the depth of anaesthesia monitoring in China. DOI: 10.1186/s12871-021-01510-7 reasearch on specific type of technology
10. An international survey on AI in radiology in 1041 radiologists and radiology residents part 2: expectations, hurdles to implementation, and education. DOI: 10.1007/s00330-021-07782-4 - duplicate with full-text point 43
11. Knowledge, attitude, and practice of artificial intelligence among doctors and medical students in Pakistan: A cross-sectional online survey. DOI: 10.1016/j.amsu.2022.103493 duplicate with full-text point 45
12. Acceptance and Perception of Artificial Intelligence Usability in Eye Care (APPRAISE) for Ophthalmologists: A Multinational Perspective. DOI: 10.3389/fmed.2022.875242 – reasearch on specific type of technology
13. Factors Influencing Clinician Trust in Predictive Clinical Decision Support Systems for In-Hospital Deterioration: Qualitative Descriptive Study. DOI: 10.2196/33960 – reasearch on specific type of technology
14. A clinician survey of using speech recognition for clinical documentation in the electronic health record. DOI: 10.1016/j.ijmedinf.2019.07.017 – reasearch on specific type of technology
15. Investigating the Barriers to Physician Adoption of an Artificial Intelligence- Based Decision Support System in Emergency Care: An Interpretative Qualitative Study. DOI: 10.3233/SHTI200312 reasearch on specific type of technology
16. Clinician Preimplementation Perspectives of a Decision-Support Tool for the Prediction of Cardiac Arrhythmia Based on Machine Learning: Near-Live Feasibility and Qualitative Study. DOI: 10.2196/26964 reasearch on specific type of technology
17. Swedish Primary Care Physicians' Intentions to Use Telemedicine: A Survey Using a New Questionnaire - Physician Attitudes and Intentions to Use Telemedicine (PAIT). DOI: 10.2147/IJGM.S319497 – reasearch on specific type of technology
18. Attitudes towards Trusting Artificial Intelligence Insights and Factors to Prevent the Passive Adherence of GPs: A Pilot Study. DOI: 10.3390/jcm10143101 reasearch on specific type of technology
19. The medical profession transformed by artificial intelligence: Qualitative study. DOI: 10.1177/20552076221143903 researach on different group of responders
20. Otolaryngologist perceptions of AI-based sinus CT interpretation. DOI: 10.1016/j.amjoto.2023.103932 reasearch on specific type of technology
21. Undergraduate Medical Students' and Interns' Knowledge and Perception of Artificial Intelligence in Medicine. DOI: 10.2147/AMEP.S368519 researach on different group of responders
