# Supplementary material for: Physicians and AI in healthcare: insights from a mixed-methods study in Poland on adoption and challenges
Source: Front Digit Health. 2025 Mar 14;7:1556921. doi: 10.3389/fdgth.2025.1556921 (PMC11949901; doi:10.3389/fdgth.2025.1556921)
Supplement: Supplementary file 3 [file Datasheet2.pdf]

Survey – English version

Physicians' Preferences for Using AI-Based Technologies

The following survey aims to gather information about the preferences of physicians from various specialties regarding the use of AI-based technologies. The results of this survey will be used by the AIvalue4health research team to prepare the report titled *“Guidelines for Evaluating the Value of Artificial Intelligence in Healthcare in Poland”* and will contribute to a doctoral dissertation on *“The Autonomy of the Medical Profession in the Context of Implementing AI-Based Healthcare Technologies.”*

For the purposes of this survey, AI-based healthcare technologies are defined as Artificial Intelligence (AI).

---

1. How old are you?
a) <30 years
b) 31–40 years
c) 41–50 years
d) 51–60 years
e) >60 years

2. Gender
a) Female
b) Male
c) Prefer not to say
d) Other (editable field)

3. How many years have you been practicing as a physician?
a) Less than 5 years
b) 5–9 years
c) 10–20 years
d) More than 20 years

4. Do you have a medical specialization?
a) Yes
b) I am currently in training for specialization
c) No

5. What is your specialization? (optional if 4a or 4b is selected)
a) Allergology
b) Pediatric Surgery
c) General Surgery
d) Internal Medicine
e) Infectious Diseases
f) Dermatology and Venereology
g) Cardiology
h) Family Medicine
i) Neurosurgery

j) Neurology
k) Ophthalmology
l) Oncology
m) Orthopedics and Trauma Surgery
n) Otorhinolaryngology
o) Pediatrics
p) Obstetrics and Gynecology
q) Psychiatry
r) Radiology and Imaging Diagnostics
s) Urology
t) Other (editable field)

6. What is your primary place of work?
a) Pharmaceutical industry
b) Private clinic
c) Public clinic
d) Private hospital
e) Public hospital
f) Other (editable field)

7. Where is your primary place of work located?
a) In a city (more than 1 million inhabitants)
b) In a city (500,000–1 million inhabitants)
c) In a city (50,000–500,000 inhabitants)
d) In a city (up to 50,000 inhabitants)
e) In a rural area

8. Do you use AI in your daily practice?
a) Yes
b) No, and I do not plan to use AI in my work in the future
c) No, but I am open to using AI in my work
d) I don't know

9. Do you think AI can replace a physician's work?
a) Yes, within the next 20 years
b) Yes, but it is a distant future (more than 20 years)
c) No, but the role and responsibilities of a physician will change
d) No, but physicians using AI will replace those who do not
e) No, AI will never replace a physician's work

10. Would you be interested in training on the use of AI in healthcare?
(Please rate on a scale from 1 to 5, where 5 means "very interested" and 1 means "not interested at all.")

5 (very interested)	4	3	2	1 (not interested at all)

11. **Please rate the extent to which you agree or disagree with the following statements. Remember, there are no right or wrong answers**

	Strongly agree	Somewhat agree	Neutral	Somewhat disagree	Strongly disagree
The use of AI in healthcare will reduce the number of diagnostic errors.					
AI-based decision support systems are tools that enhance the efficiency of a physician's work.					
Thanks to the use of AI, physicians will have more time for their patients.					
The use of AI-based technology will negatively impact doctor-patient relationships					
Physicians should only be allowed to use AI after completing specialized training in this area.					
Legal accountability for decisions recommended by AI should be assigned to the technology producer.					
Regular validation and reliability checks of AI are essential to enable its use.					
Atypical medical cases cannot be diagnosed using AI.					
The use of AI carries the risk of improper use of patient personal data.					
Every AI system should have scientific evidence of beneficial effects before being implemented for use.					

12. In my opinion, the greatest benefits of using AI by physicians are:
(Rank the answers in order, with the greatest benefit at the top and the least benefit at the bottom)

a) Easier access to healthcare services for patients
b) More efficient diagnostics and clinical decision support for physicians
c) Reduced healthcare costs
d) Fewer administrative tasks performed by physicians
e) More precise and less invasive procedures

13. In my opinion, the risks associated with the use of AI by physicians are:
(Rank the answers in order, with the greatest risk at the top and the least risk at the bottom)

a) Reduced doctor-patient interaction
b) Risk of disclosing patient personal data
c) Inaccuracy of diagnosis and errors in recommendations generated by AI-based technology
d) Job loss and/or changes in the scope of physicians' responsibilities
e) Ethical and legal issues related to accountability for incorrect recommendations generated by AI-based technology
f) Requirement for training in the use of AI-based technologies
